# Socioenvironmental factors associated with *Schistosoma mansoni* infection and intermediate hosts in an urban area of northeastern Brazil

**DOI:** 10.1371/journal.pone.0195519

**Published:** 2018-05-02

**Authors:** Taíssa Alice Soledade Calasans, Geza Thais Rangel Souza, Claudia Moura Melo, Rubens Riscala Madi, Verónica de Lourdes Sierpe Jeraldo

**Affiliations:** 1 Laboratory of Infectious and Parasitic Diseases, Institute of Technology and Research (Instituto de Tecnologia e Pesquisa—ITP), Aracaju, Sergipe, Brazil; 2 Graduate Program in Health and Environment, Tiradentes University (Universidade Tiradentes), Aracaju, Sergipe, Brazil; 3 Tropical Biology Laboratory, Institute of Technology and Research, Aracaju, Sergipe, Brazil; Centers for Disease Control and Prevention, UNITED STATES

## Abstract

Schistosomiasis, which is caused by trematodes of the genus *Schistosoma* and by the species *Schistosoma mansoni* in Brazil, is transmitted primarily by *Biomphalaria glabrata* mollusks. Infections occur in humans and mollusks in freshwater environments contaminated with feces from infected humans. This study aimed to evaluate potential foci of schistosomiasis based on the identification of infection sites for the snails, factors that increased the human infection probability of *S*. *mansoni* infection, and the relationship of the disease with abiotic, biotic, and sociocultural factors. The study was conducted in an urban area on the northeast coast of Brazil; this location was chosen based on the following factors: the presence of *B*. *glabrata*, nearby freshwater, and the absence of sewer treatment. A parasitological analysis was performed to evaluate infections of the mollusks and residents inside the perimeter defined by the collection points. Questionnaires were applied to obtain demographic data and to identify behaviors that led to human infection. To verify the contamination of freshwater by human feces, a microbiological analysis of the water was performed at the mollusk collection points to determine the rate of contamination with fecal coliforms. A total of 10,270 *B*. *glabrata* mollusks were collected between August 2013 and August 2014, of which 8.8% were positive for *S*. *mansoni*; the prevalence ranged from 0 to 34.5% over the study period. A total of 232 coprological samples from the residents were analyzed. The *S*. *mansoni* infection prevalence rate was 16.4%, and the *S*. *mansoni* parasitic load in the infected residents was 54.9 eggs per gram of feces on average. Males were more affected by the parasite, especially in the 8-17-year-old age range. Thermotolerant coliforms were observed at the mollusk collection sites, which indicated that freshwater and sewage were in continuous contact. This contamination indicated poor sanitary conditions, as was previously observed, which could be combined with detrimental behavior due to the residents' habits. These conditions cause a predisposition for both intermediate and definitive infections of the hosts by creating a socioenvironmental scenario that is conducive to the formation and maintenance of potential schistosomiasis foci. This and similar areas deserve special attention from the government with an aim of improving sanitation services and local resident knowledge to prevent future contamination.

## Introduction

Schistosomiasis is considered a neglected parasitic disease and affects over 200 million people worldwide.This disease is caused by trematodes of the genus *Schistosoma* (Weiland, 1858) [1] and by the species *Schistosoma mansoni* in Brazil (Sambon 1907). In Brazil, *Biomphalaria glabrata* (Say, 1818) is one of the most important intermediate host species of *S*. *mansoni* due to its wide geographic distribution and high infection rates. The occurrence and spread of schistosomiasis are related to poor environmental conditions, with a high prevalence of human cases usually found in people live in unfavorable socioeconomic conditions [2,3,4].

Human and mollusk infections occur in freshwater bodies contaminated with the feces of people infected with schistosomes. Absent or inadequate sanitation, human cultural habits, and the presence of the *Biomphalaria* mollusk contribute to the persistence of the parasite's life cycle and consequently to the disease's geographic spread [5,6,7]. In Brazil, *S*. *mansoni* infection is considered a public health problem in 19 states. As a result, Brazil is considered the Latin American country with the greatest transmission foci, with approximately 30 million people at risk of contracting the disease and approximately 4–6 million people already infected [8,9,10].

Initially, schistosomiasis was typically endemic in rural areas; however, due to human migration, transmission also occurs in large Brazilian urban centers, with the northeast of the country having the highest prevalence rates [9, 11]. For instance, the state of Sergipe had a prevalence of 13.6% (14,471 positive cases/106,287 people analyzed) in 2005, 11.2% (16,196/145,069) in 2006, 11.8% (10,220/86,824) in 2007, and 10.6% (8,329/78,859) in 2008 based on parasitological examinations performed by the Brazilian Schistosomiasis Combat Program (Programa de Combate a Esquistossomose–PCE). In each year, 100% of the municipalities analyzed tested positive for *S*. *mansoni* infections [12]. Moreover, the tropical climate contributes to the establishment of schistosomiasis in this region because it provides conditions suitable for the presence of the mollusks that encourage the transmission of this endemic disease when associated with poor sanitary conditions [13].

Neglected tropical diseases, such as schistosomiasis, require prevention and control measures and elimination efforts; therefore, an understanding of the transmission factors in a particular area should be obtained [14,15]. These factors include the characteristics of the environment and the sociocultural processes that influence the transmission process. Moreover, implementation of health education interventions, preventive chemotherapy directed at the entire community, and access to sanitation and safe water are essential to interrupt the transmission and control cycle of schistosomiasis [16]. Furthermore, identifying the breeding grounds of the *S*. *mansoni* mollusk intermediate host and performing an evaluation of its ecological and biological aspects are critical for investigations of the transmission risk rates and the implementation of control strategies in locations where the disease is established [17,18].

The aim of this study was to evaluate potential schistosomiasis foci based on studies on the prevalence of *S*. *mansoni* infection in mollusks and individuals who lived near these foci, the influence of rainfall and environmental contamination due to the lack of a proper sanitation system, and sociocultural aspects of this endemic disease’s transmission process in an urban community in northeastern Brazil.

## Materials and methods

### Study site and population

**Fig 1:**
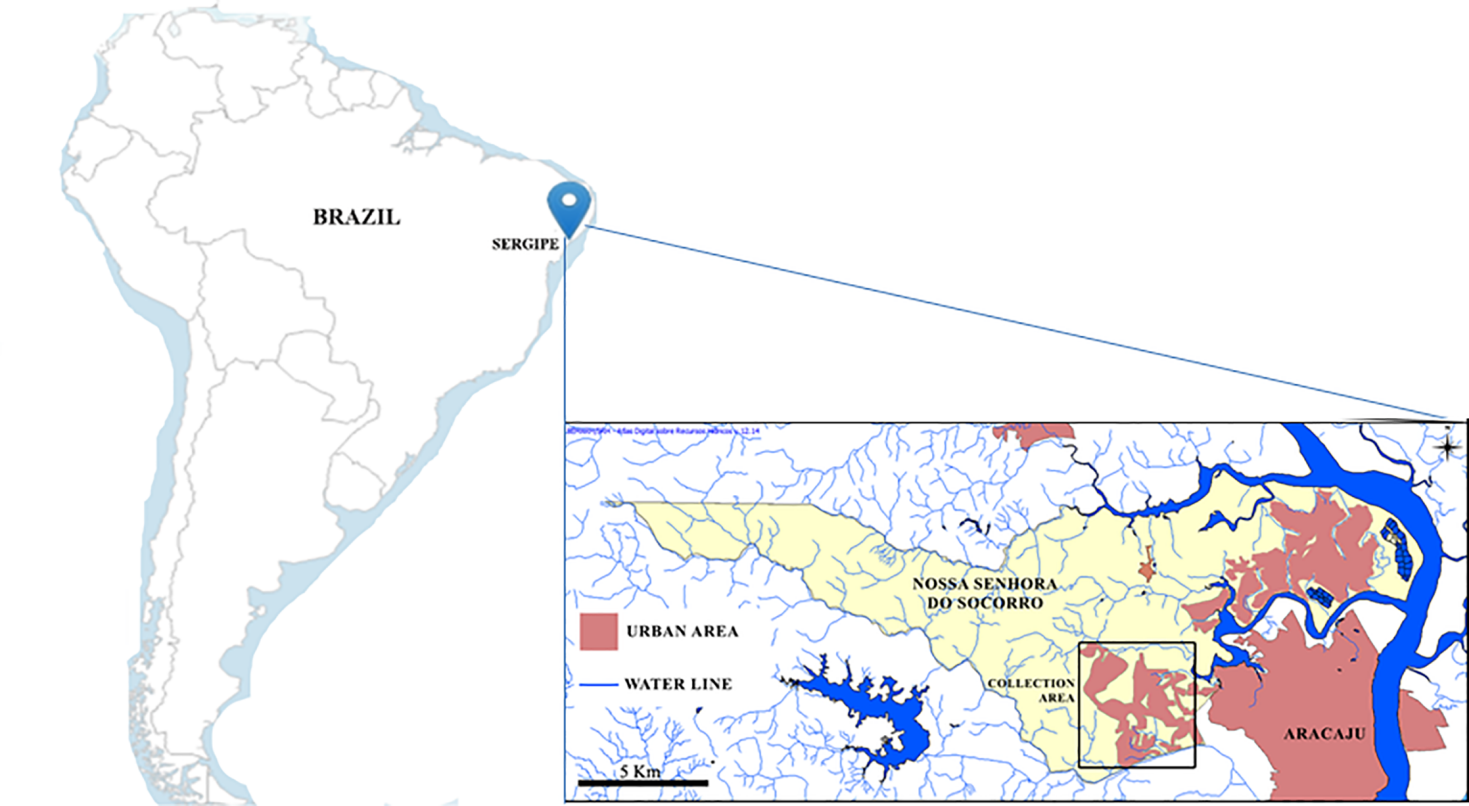
Location of the state of Sergipe and the Nossa Senhora do Socorro municipality showing the traveled perimeter in northeastern Brazil (Source: Atlas Digital- Quantum Gis 1.7.0).

The study was conducted in a community in the city of Nossa Senhora do Socorro, which is located in the state of Sergipe. The city has approximately 155,334 inhabitants and a land area of 158 km^2^ in northeastern Brazil. The community named Parque dos Farois has a population of approximately 15,000 according to local health service information. The study area has a biome characterized by Atlantic forest and a hot and humid tropical climate and is located on the shores of the sub-basin of the Poxim River (Fig 1). The main environmental issues affecting the study area are riparian deforestation, trash in the open, disordered urban occupation, and deficiencies in the water supply and sanitary network [19].

### Ethical aspects

The study received institutional approval from the Ethics Committee on Human Research from Tiradentes University under protocol log number 28689514.4.0000.5371. All participants were invited to sign the free and informed consent term. In this document, participants must have the freedom to leave the study at any time. All questions are clarified to ensure that there is no doubt about your participation without study. In cases of illiteracy, a thumbprint and a signature of a witness were collected. The parents or guardians signed the consent form authorizing the participation of minors under 18 years of age. By signing the form, the participants agreed to participate in the interview through questionnaires and to provide a fecal sample for the parasitological analysis. Participants under 18 years of age were assisted by parents or guardians during the interview. The results were communicated to the participants, and adults and children under 18 years of age infected with any type of helminth were referred to the local health unit for treatment with 50 mg/kg body weight of praziquantel (Farmanguinhos® 600 mg; manufactured by the Institute of Technology in Pharmaceuticals/Farmanguinhos with registration by the Oswaldo Cruz Foundation). The treatment accordance with according to the Brazilian guidelines of the Control Program of Schistosomiasis (PCE).

### Rainfall data

The data related to rainfall were obtained from the Department of Water Resources of the State of Sergipe (Secretaria de Recursos Hídricos do Estado de Sergipe—SRH-SE) for monthly correlations with the prevalence and abundance of the collected mollusks.

### Mollusk collection and cercarial emission

Mollusks were collected at five georeferenced points identified as P1 (10°55′230′′S 37°8′161′′W), P2 (10°55′231′′S 37°8′135′′W), P3 (10°55′326′′S 37°8′00′′W), P4 (10°55′18.37′′S 37°7′56.98′′W), and P5 (10°55′16.04′′S 37°7′51.80′′W), which were distributed in streams located near residences. The points were selected based on work that developed for the identification of mollusk transmitters of schistosomiasis by health workers operating in the Schistosomiasis Control Program (PCE) operated by the state government.

Mollusks were collected with the aid of sieves and tongs. Each person conducted collections for a 1-hour period monthly between August 2013 and August 2014. *S*. *mansoni* infection was evaluated by individual exposure of the mollusks to artificial light at a distance of 30 cm for 4 hours. This procedure provided a temperature between 28 and 30°C, which was capable of stimulating cercarial emission [20, 21].

### Questionnaire survey

To evaluate human infection with *S*. *mansoni*, a non-probabilistic, cross-sectional study was performed for accessibility in a 0.64-km^2^ area where mollusk collections were conducted concomitantly during the study period. The residents of all households located approximately 4.5 km from this area were visited and invited to participate in the study regardless of their ages gender, and whether or not they had a diagnosis of *S*. *mansoni* infection.

A questionnaire was used to interview the participants face to face. The questionnaire consisted of multiple choice questions or binary answers, such as affirmation and negation questions (yes or no), and was used to obtain information about individual patterns divided into the following categories: family identification data (address, age, gender, place of birth, and residence time in the area), economic data (family income), and environmental/cultural aspects (water supply, garbage disposal, sewage, and forms of contact with the river/stream water, among other information) (S1 Appendix).

### Human parasitological diagnosis

Following the principles of non-probabilistic sampling for convenience, all individuals who participated in the interviews received a plastic container for the collection of feces. The containers were collected, identified, and transferred to the laboratory for the parasitological analysis. One sample was collected from each individual in the morning in 2014. Two slides were prepared using the qualitative spontaneous sedimentation technique and the quantitative Kato-Katz technique. The Kato-Katz technique was also used to determine the parasitic load of the individuals for *S*. *mansoni*. The parasite load was defined by the number of eggs found on the slides and multiplied by the constant 24 and thus the number of eggs per gram of faeces (opg) was obtained, and was categorized as low light (1–99 epg), moderate (100–399 epg), or heavy (≥400 epg) [16, 22]. Individuals who had *S*. *mansoni* eggs on at least one of the two analyzed slides were considered positive. Other parasites found in the analyses were also included in this study.

### Microbiological analysis

To determine the presence of total and thermotolerant coliforms, 100 ml of water was collected from points P1, P2, P3, P4, and P5 in both the dry and rainy seasons of 2014. The multiple-tube quantitative method was applied for the microbiological evaluation to determine the “most probable number” (NMP) of target microorganisms in the sample through the distribution of aliquots in a series of tubes containing differential culture media for the growth of the target microorganisms [23]. The sodium lauryl sulfate, Brilliant Green, and EC broths were used for the analysis. In the sodium lauryl sulfate broth, the presumed presence of coliforms was evidenced by the formation of gas in the Durham tubes, which was produced by the fermentation of lactose present in the environment. The Brilliant Green broth was used for confirmative evidence of total coliforms in the confirmed positive tubes inoculated in the sodium lauryl sulfate broth [24].

The total and thermotolerant coliform levels were analyzed and compared to the limits set by resolution 357 of the National Environmental Council (Conselho Nacional do Meio Ambiente—CONAMA) from March 2005, which provided the designation "Class 3 fresh water" to designate water suitable for human consumption.

### Statistical analysis

The software STATISTICA 7.1 was used to perform the Spearman test correlation (rs) was used between two quantitative variables. This test was applied between the average of the monthly rainfall and the abundance and infection of mollusks. O software BioEstat 5.3 was used to perform the for the analysis of the independent variables and their associations with people with or without schistosomiasis, odds ratios were calculated (OR) with 95% confidence intervals (CIs) to verify the influence on the occurrence of a certain event between the parasitized and non-parasitized groups and Student’s paired t-test was used to compare the microbiological parameters of the NMP to the values established by the CONAMA resolution. A 5% level of significance was adopted for all analyses [25].

## Results

### Malacological study

**Fig 2:**
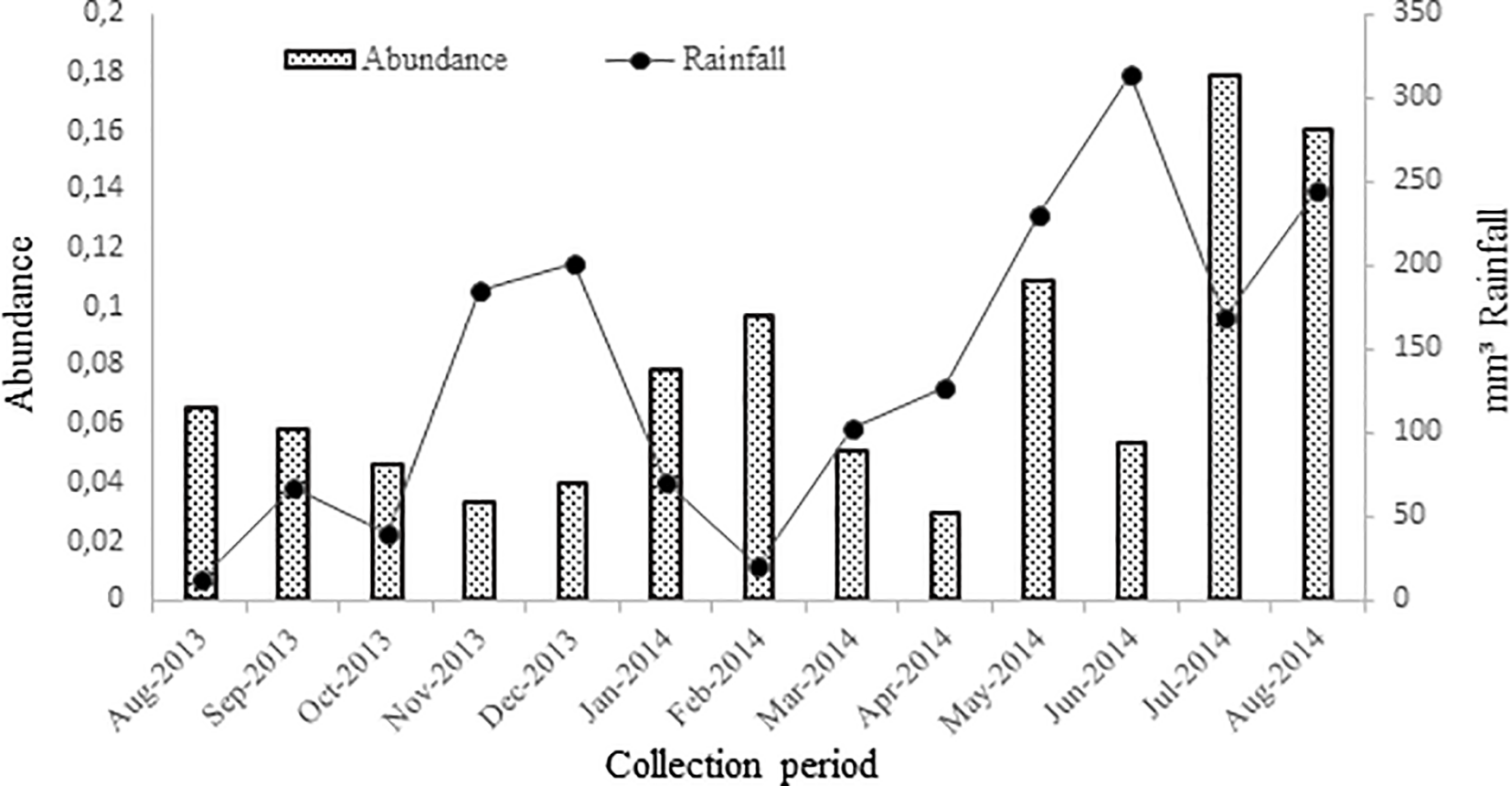
Distribution of *Biomphalaria glabrata* snails collected in urban areas and the monthly precipitation index. Nossa Senhora do Socorro municipality, Sergipe, Brazil.

Fig 2 shows the distribution of the collected mollusks. A total of 10,270 *B*. *glabrata* mollusks were collected during the study period. There was a negative correlation [(rs) = -0.03, n = 10.270, p> 0.05] between a monthly average rainfall and an abundance of *B*. *glabrata* occurring in the previous season (one month before). Mollusks were collected 2,487 (IC 95%: 0.024–0.038) in the year 2013 and in the year 2014 7,883 (95% CI: 0,101–0,115).

**Fig 3:**
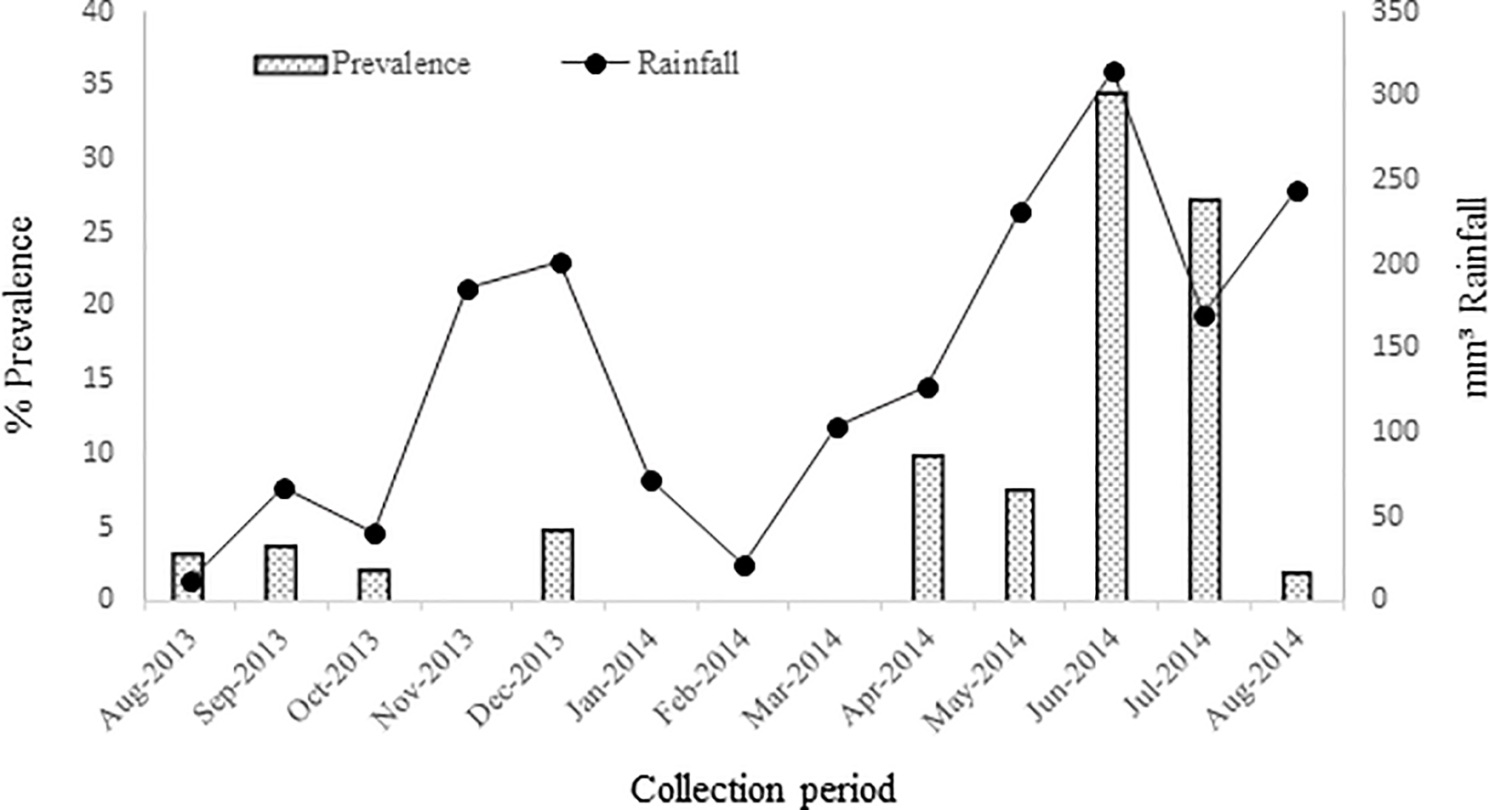
Correlation between *B*. *glabrata* infection and monthly rainfall (mm^3^) during the period from August 2013 to August 2014.

After exposure to the light stimulus to release the cercariae, 8.8% (n = 912) (95% CI: 0.083–0.094) of the mollusks were found to be positive for *S*. *mansoni*. However, the prevalence varied between 0 and 34.5% during the study period. No positive mollusks were found between November 2013 and March 2014 (with the exception of December); however, starting in April, mollusks positive for *S*. *mansoni* infection were found coinciding with the rainy season (Fig 3). A correlation was observed between rainfall and *B*. *glabrata* infection during the study period [(rs) = 0.042, n = 10.270 p<0.05].

### Human infection

Of the 232 stool samples collected from individuals living close to the mollusk collection points, 66.4% had at least one intestinal parasite. *Schistosoma mansoni* infection had the second highest prevalence at 16.4% when the spontaneous sedimentation and Kato-Katz techniques were combined (Table 1). The *S*. *mansoni* parasitic load of the infected population was 54.9, with a range from 24 to 144 eggs per gram (epg) of feces.

**Table 1:** Prevalence of *S*. *mansoni* and intestinal parasitic infections determined by the spontaneous sedimentation and Kato-Katz methods.

Infeccion by	Number (%)						
	Spontaneous Sedimentation	Kato—Katz	Combined results*				
	method (n = 232)	method (n = 232)	Men (n = 89)	Women (n = 65)		boths sexes (n = 232)	95.0% C.I.# for OR
***S*. *mansoni***	31 (13.4)	7 (3.1)	22 (24.7)	16 (24.6)	38 (16.4)		0.12–0.22
***A*. *lumbricoides***	30 (12.9)	34 (14.6)	40 (44.9)	24 (36.9)	64 (27.6)		0.22–0.24
***T*. *trichiura***	10 (4.3)	11 (4.7)	13 (14.6)	8 (13.3)	21 (9.1)		0.06–0.13
**Hookworm**	10 (4.3)	0 (0)	7 (7.8)	3 (4.6)	10 (4.3)		0.02–0.08
***E*. *vermicularis***	2 (0.8)	4 (1.7)	3 (3.4)	3 (4.6)	6 (2.6)		0.01–0.06
***H*. *nana***	2 (0.8)	0 (0)	0 (0)	2 (3.1)	2 (0.8)		0.00–0.03
***E*. *coli***	10 (4.3)	-	3 (3.4)	7 (10.7)	10 (4.3)		0.02–0.08
***E*. *histolytica/dispar***	1 (0.4)	-	1 (1.2)	0 (0)	1 (0.4)		0.00–0.02
***G*. *lamblia***	2 (0.8)	-	0 (0)	2 (3.1)	2 (0.8)		0.00–0.03

*Combined result indicates any people that was positive either by Spontaneus Sedimentation or Kato-Katz.

#Confidence Intervals 95.0% (C.I.95%).

Individuals infected with other helminths were encountered, particularly *Ascaris lumbricoides* with a prevalence of 27.6%. The commensal *Entamoeba coli* was among the most prevalent protozoans (Table 1).

**Fig 4:**
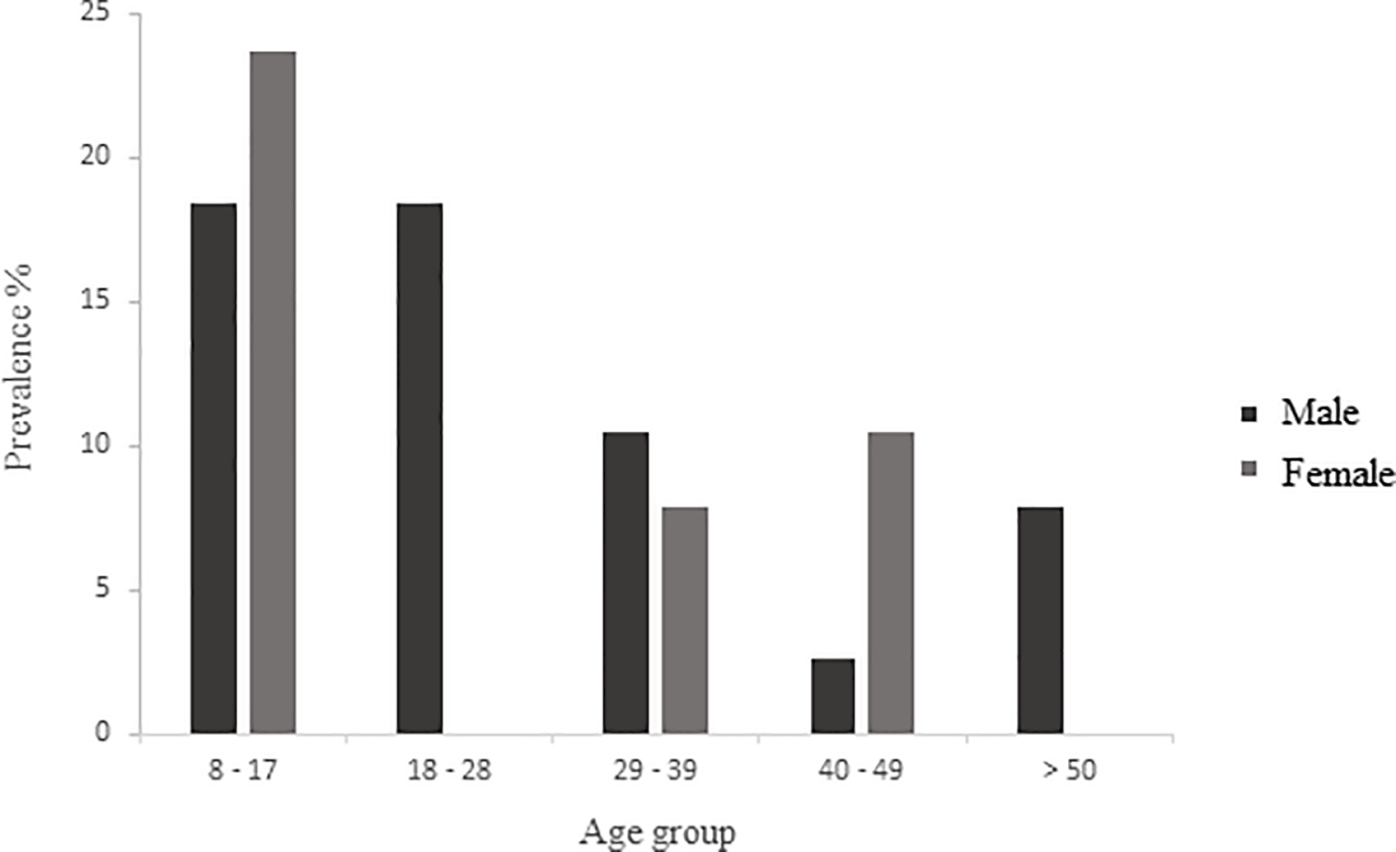
Age group distribution of *S*. *mansoni* infection, 2014.

Regarding the age group, the majority of individuals infected with *S*. *mansoni* were children and adolescents in the 8- to 17-year-old age group and were represented by both genders (18.4% males and 23.7% females) (95% CI: 0.279–0.578). No differences were observed in the prevalence rates between men (10.5%) and women (7.9%) in the 29- to 49-year-old age group (95% CI: 0.191–0.475). Only males were infected in the age group of 18–28 years (18.4%) (95% CI: 0.092–0.334). However, only males were infected (7.9%) in the 50 years and above age group (95% CI: 0.027–0.208). Generally, *S*. *mansoni* infection was more prevalent in males, with the exception of the 40- to 49-year-old age group (95% CI: 0.058–0.273) (Fig 4).

### Questionnaire survey

#### Demographic and socioeconomic variables

Regarding provenance, 65.8% of the residents infected with *S*. *mansoni* came from the state of Sergipe, 13.1% from Alagoas, and 7.9% from Pernambuco. In Table 2 it is possible to check the residence time of infected residents, and 50% reported living in place of 2 to 10 years and 36.8% live 11 to 20 years. The level of education of individuals infected with *S*. *mansoni* stood basically on incomplete primary education, with 78.9% of the population that low education level. The socioeconomic approach showed that 89.5% of the population had a low wage band to a minimum income.

**Table 2:** Residence time of individuals infected with Schistosoma mansoni in a community of Nossa Senhora do Socorro municipality, Sergipe, Brazil—2014.

Residence time	Individuals	
	*N	%
**2 a 10 years**	19	50
**11 a 20 years**	14	36.8
**> 20 years**	5	13.2
**Total**	38	100

*N = Number

#### Risk factors for *S*. *mansoni* infection

The odds ratio analysis for each of the variables tested showed that male residents were 3.86 times more likely to be infected with *S*. *mansoni*. The remaining analyzed variables showed no significant risk of infection (Table 3). However, the area has poor sanitation, with residential waste dumped directly onto the streets through plumbing pipes improvised by the residents themselves, leading to the presence of open sewers with streams and puddles formed by both rainwater and residential sewage. These streams and puddles contained mollusks infected with *S*. *mansoni*.

**Table 3:** Association of environmental and demographic factors with *Schistosoma mansoni* infection in residents of the Nossa Senhora do Socorro municipality, 2014.

Risk Factors	*S*. *mansoni*				OR*	95.0% C.I.^#^ for OR	P-Value
	Positive		Negative				
**Potable water**	Yes	10		81	0.84	0.35–2.00	0.873
	No	14		96			
**Garbage collection**	Yes	22		153	0.62	0.16–2.36	0.7534
	No	3		13			
**Sewerage**	Yes	13		92	0.93	0.40–2.15	0.9633
	No	12		79			
**Rudimentary cesspool**	Yes	15		122	0.72	0.27–1.88	0.6816
	No	7		41			
**Contact (water streams)**	Yes	13		79	1.22	0.51–2.89	0.8048
	No	11		82			
**Contact (river water)**	Yes	15		93	1.19	0.50–2.81	0.8500
	No	10		74			
**Habit (Barefoot)**	Yes	14		86	0.96	0.41–2.25	0.8975
	No	11		65			
**Sex**	M	29		131	3.86	1.76–8.44	<0.0007
	F	9		157			

*OR = Odds Ratio

#Confidence Intervals 95.0% (C.I.95%)

#### Microbiological water analysis

The microbiological analysis results revealed different levels of contamination by total and thermotolerant coliforms in the samples from the five mollusk collection points. During the dry season, the total coliforms ranged from 2.1 x 10^4^ to 7.5 x 10^4^, and the fecal coliforms/thermotolerant coliforms ranged from 1.4 x 10^3^ and 4.6 x 10^3^. During the rainy season, the total coliforms ranged from 1.7 x 10^4^ to 4.7 x 10^4^, and the fecal coliforms/thermotolerant coliforms ranged from 1.4 x 10^3^ to 5.4 x 10^3^. During both the dry and rainy seasons, the thermotolerant coliform concentration was in accordance with the CONAMA resolution and showed no significant differences when the total mean was compared with the limits set by CONAMA. Conversely, the total coliform levels were above the allowed limit, and the paired t-test revealed total mean values significantly above those set by CONAMA in both evaluation periods: dry season—p < 0.0322, mean 3.54, and standard deviation ± 2.24; rainy season—p < 0.0094, mean 3.12, and standard deviation ± 1.24.

## Discussion

Epidemiological studies of schistosomiasis are important for elucidating the dynamics of transmission in endemic areas because each site has biological, ecological, social, and economic characteristics that may affect this process [26]. The urban area studied presents a social and environmental scenario that is conducive to the formation/maintenance of potential schistosomiasis transmission foci. The presence of mollusks in the water bodies, the individual cultural habits of each resident, and the precarious nature of the basic sanitation are essential for understanding the results obtained in this study.

In the scenario of schistosomiasis transmission in Brazil, *B*. *glabrata* mollusks are important actors because of their wide geographic distribution and the high rates of S. mansoni infection reported by other authors [11,12]. In the study area, the parasitological analysis revealed the presence of *B*. *glabrata* mollusks releasing *S*. *mansoni* cercariae, (8.8%), which can be considered as a sign of the presence of transmission focus ([27]) in view of the fact that infected residents were also found (16.4%).

On the other hand, the Schistosoma transmission has an important seasonal component that is related to the distribution and density of the intermediate host and determined in the rainy periods by the increase of the rivers flow and the temporary water pools [28]. Although this study revealed no significant association between the mollusk density and rainfall, the latter factor appeared to influence these parameters; indeed, the density of the mollusks present and the mollusks shedding eliminating *S*. *mansoni* cercariae increased as the rainfall time increased. This correlation has been reported in other studies conducted in Brazil, which is the main endemic area for mansonic schistosomiasis in the Americas because the hot and humid tropical climate is conducive to the reproduction and maintenance of the host mollusks [8,29,30]. Before the rainy season, an important measure for the control of schistosomiasis is to maintain the cleanliness of the urban environment, as this can promote a significant reduction in the number of mollusks and their potential dispersion during the rainy season.

The identification of schistosomiasis transmission foci is crucial for the development of control and health management plans in these communities [31]. Based on the location of mollusks infected with *S*. *mansoni*, we performed a parasitic evaluation of the community close to the mollusk collection sites. The results showed prevalence greater than 15%; thus, the area can be considered highly prevalent even though the intensity of the infections was low, with less than 100 eggs per gram of stool [16]. Similar to reports in other studies [32], this fall in the infection rate is the result of control measures based only on treatment without the implementation of any other major interventions in the health infrastructure [33].

A cross-sectional study conducted in the same area but based on data from the Schistosomiasis Control Program (Programa de Controle da Esquistossomose—PCE) from 2003–2008 found that the municipality of Nossa Senhora do Socorro was considered to have medium endemicity, with a mean prevalence of 13.98% [31]. These results show that the epidemiological status of schistosomiasis has undergone no major changes in improvements in the population over the last 10 years despite the efforts of government control programs. Other soil-borne parasitic infections were also identified in the residents of the study area, showing that the population was exposed to various infectious agents probably due to the poor sanitary conditions in the area.

The highest prevalence encountered in this study occurred in children and adolescents, which corroborated the results from other studies that identified these age groups as the most affected due to the lack of hygiene, more frequent contact of young people with contaminated water, and their recreational activities. The greatest number of positive cases in Brazil has been found in the 6- to 20-year-old age group [6, 33, 34].

Together with the state of Sergipe, other states of the Brazilian Northeast are considered endemic for schistosomiasis, which reinforces the attention to the spread of the disease in the region and also to other regions of Brazil and the world as a result of migratory processes [6,9,32,33]. Many of the individuals infected with *S*. *mansoni* in this study were born in other Northeastern states such as Alagoas and Pernambuco which may also contribute to the spread and establishment of endemic disease in the study area. Notably, all positive individuals had a low education level and family income, reinforcing the fact that schistosomiasis is strongly linked to a low socioeconomic status.

Several studies have reported that schistosomiasis is a disease that affects males more frequently [35, 36], which was also observed in our study, which showed that these have a significantly higher chance of acquiring the infection. This result may be related to the greater involvement of men in activities in rivers and streams near the community, such as fishing, river bathing for leisure and other activities. Similar results were found in the literature for studies conducted in the northeastern region of Brazil that showed higher prevalence rates for *S*. *mansoni* in males; the studies also suggested that the reason for these higher prevalence rates was possibly because males attended health services less frequently [37, 38, 39].

Although there was no significant association between schistosomiasis and demographic, social, environmental and cultural variables, certainly constant contact in areas with poor sanitation and in which infected intermediary hosts were detected may have contributed to the transmission of *S*. *mansoni* in the locality. Additionally, contact with contaminated water can also be accidental in the rainy season, when floods and overflowing streams facilitate human contact with the parasite [40, 41]. The same variables, sewage destination and water supply were analyzed in a study carried out in the State of Alagoas/ razil, in which no significant associations with *S*. *mansoni* infection were found [6].

Microbiological water quality evaluation is an essential tool for the prevention of waterborne infections. In addition to indicating the fecal contamination rate, this analysis is relevant because it focuses on confirming the presence of human waste in the water from which *B*. *glabrata* was collected. This finding is of interest because mollusk and human infections are strongly linked to contamination of the aquatic environment by coliforms [42]. The results obtained in this research revealed that the freshwater bodies were contaminated with total and thermotolerant coliforms, which certainly confirmed sewage discharge in that area and increased the likelihood that the *S*. *mansoni* eggs came from infected individuals in the community. Coliform bacteria have been extensively used as fecal pollution indicators in the evaluation of the sanitary conditions of water bodies because these microorganisms are specific to the human intestinal tract [32].

This study reveals worrying data regarding schistosomiasis in the community, including high prevalence of infection, presence of *S*. *mansoni* infected mollusks in streams located in front of residents' homes. These findings point to the need to implement comprehensive control measures aimed at controlling the population of mollusks because residents share the urban space with the residents, associated with improved sanitation conditions so that the sewage is not dumped directly into the environment.

The situation of the urban community under study shows a new epidemiological situation observed in many Brazilian cities, the disorderly growth of cities often caused by rural migration, which gives rise to pockets of poverty in which residents live without adequate sanitation conditions being exposed to diverse diseases such as schistosomiasis. The Government's program of disease control in Brazil has worked and undoubtedly achieved good overall results, however this new urban transmission profile must be investigated and recognized in its particularities since each place has different socioenvironmental characteristics.

In conclusion, the prevalence of schistosomiasis in the studied community is related to the aforementioned conditions that contribute to the maintenance of the endemic cycle. These results indicate that understanding the peculiarities of endemic areas is important for defining specific prevention and control strategies. Government intervention is necessary in the actions of improvements in sanitation and the treatment of infected residents with an aim of disrupting the biological cycle of *S*. *mansoni* to attempt to minimize the risks of transmission and the emergence of new cases.

On the other hand, the recognition by the population of the need for health care is fundamental to reverse situations such as the one observed in the present research and that certainly go through the implementation of educational actions. Individuals infected with *S*. *mansoni* and/or other parasites were referred to the local health clinic for treatment, which does not guarantee that they will be reinfected in the same place. Finally, the data presented in this research show the reality of several urban communities concentrated in the periphery of many Brazilian cities and which clearly show that schistosomiasis in addition to other health situations could be minimized in a relevant way if, for example, adequate basic sanitation was offered to these populations.

## Supporting information

S1 Appendix Questionnaire used in interviews with residents to obtain the results of socioeconomic and demographic variables. In addition to risk factors for *S*. *mansoni* infection.
(PDF)
