# Supplementary material for: Socioenvironmental factors associated with Schistosoma mansoni infection and intermediate hosts in an urban area of northeastern Brazil
Source: PLoS One. 2018 May 2;13(5):e0195519. doi: 10.1371/journal.pone.0195519 (PMC5931446; doi:10.1371/journal.pone.0195519)
Supplement: S1 Appendix — In addition to risk factors for S. mansoni infection. (PDF) [file pone.0195519.s001.pdf]

PROFILE OF EPIDEMIOLOGICAL Schistosomiasis in an endemic area in municipality of Nossa Senhora do Socorro
Questionnaire 1 - ENVIRONMENTAL, economic, and cultural population's health of schistosomiasis

GENERAL DATA

Date: ___/_____/_______ Address:____

- **IDENTIFICATION INFO**:

1) Name (Initial): ______________________ 2) Gender: () F () F

3) Address: ___________________________________ Complement: _____________

4) Date of Birth: ___ / ________ / ______ (dd / mm / yyyy) Age: ________

5) Race:
() White () Black () Brow () Indigenous () East

6) Birth: _________________ State: __________

7) Status:
Single () Married () Divorced () Widowed () Stable Union ()

8) Residence time in the headlights Park Set:
Years: ________ Meses_________
Obs .: If <1 year specify place of origin: ______________

- **SOCIOECONOMIC DATA**:

9) This working:
( ) Yes No

10) What is your main activity: ______________________

11) What is your status in employment
() Retired () Entrepreneur () From Home () Salaried () Student
() Self-Employed () Public Employee () Pensioner () Other ____________

12) Household Income:
() No yield; () Up to 1 MW; ( ) More1 to2 SM; ( ) More2 to 3 MW;
( ) More 3 to5 MW; () More than 5 MW.

If no reply or to yield 1Sa:
Receive any supplementary official income such as Bolsa Familia () Yes () No

13) Education:
Illiterate (); Key () Complete () Incomplete
Medium ( ) Complete ( ) Incomplete
Graduated ( ) Incomplete

- **SOCIAL AND ENVIRONMENTAL DATA**:

14) Form water supply at home:
() Public; ( ) Artesian well; () Direct collection in the river; ( ) Other

15) Water treatment at home for consumption:
( ) No treatment; () Filtration; () Boiling; () Chlorination; ( ) other___________

16) Waste Destination of the household:

() Public collection; () Burnt; () Buried; () Played in a vacant lot; () Played in the river;
() Another destination.

17) Disposition of fecal / urine household:
( ) Sewerage; () Septic; () Played in a vacant lot; () Played in the river;
() Another destination.

18) Type of house:
() Brick / adobe; () Coated Taipa; () Taipa uncoated;
( ) Wood; ( ) Board; () Material not used.

19) The floor of the house:
( ) Cement; () Ceramics; () Clay apilado.

20) You have access to electricity:
( ) Yes No

- **<u>CONTACT INFORMATION WITH RIVER WATER / STREAM / OTHER (specify) ....</u>**:

21) Have you ever had contact with river water:
( ) Yes No
If yes, how long: _____________

22) Since the contact happens:
() Car Wash / animal; () Washing; () Wash dishes / pot; ( ) Recreation; () Personal Hygiene;
() Fishing.

23) Have you had direct contact with the water streams:
( ) Yes No.
If so, why not: ______________________

24) Other (report)
....
....
....

- **<u>DISEASES ASSOCIATED DATA AND LIFE HABITS</u>**:

24) You are stricken by some (s) disease (s):
() Yes () No If yes, which one (s): _________________________

25) Have you had any signs and / or symptoms:
Diarrhea () Yes () No; Mucus in the stool () Yes () No; Blood in the stool () Yes () No

26) makes use of alcoholic beverages:
( ) Yes No

27) Do you smoke:
( ) Yes No

28) habit of eating vegetables or fruits without washing:
( ) Yes No.

29) smoker walking barefoot through the streets of your neighborhood:
( ) Yes; ( ) No.
